# Imputation for transcription factor binding predictions based on deep learning

**DOI:** 10.1371/journal.pcbi.1005403

**Published:** 2017-02-24

**Authors:** Qian Qin, Jianxing Feng

**Affiliations:** Department of Bioinformatics, School of Life Sciences and Technology, Tongji University, Shanghai, China; Ottawa University, CANADA

## Abstract

Understanding the cell-specific binding patterns of transcription factors (TFs) is fundamental to studying gene regulatory networks in biological systems, for which ChIP-seq not only provides valuable data but is also considered as the gold standard. Despite tremendous efforts from the scientific community to conduct TF ChIP-seq experiments, the available data represent only a limited percentage of ChIP-seq experiments, considering all possible combinations of TFs and cell lines. In this study, we demonstrate a method for accurately predicting cell-specific TF binding for TF-cell line combinations based on only a small fraction (4%) of the combinations using available ChIP-seq data. The proposed model, termed TFImpute, is based on a deep neural network with a multi-task learning setting to borrow information across transcription factors and cell lines. Compared with existing methods, TFImpute achieves comparable accuracy on TF-cell line combinations with ChIP-seq data; moreover, TFImpute achieves better accuracy on TF-cell line combinations without ChIP-seq data. This approach can predict cell line specific enhancer activities in K562 and HepG2 cell lines, as measured by massively parallel reporter assays, and predicts the impact of SNPs on TF binding.

## Author summary

Transcription factors play a central role in regulating various cellular processes. They bind to DNA in a cell-specific way. To study where a TF would bind to DNA, ChIP-seq experiment has been developed and widely adopted by the science community to study genome-wide in vivo protein-DNA interactions. However, for each TF, only a limited number of cell types have been explored by ChIP-seq experiments. To study the binding of a TF to a DNA sequence in a cell line without corresponding ChIP-seq data, researchers would check whether there is a motif for the TF in the sequence. However, motif alone contains only sequence information and therefore cannot reflect the cell specificity of TF binding. In this work, we demonstrate how to model the TF binding problem using deep learning and achieve cell specific binding prediction for TF-cell line combinations without ChIP-seq data.

This is a *PLOS Computational Biology* methods paper

## Introduction

Transcription factors (TFs) play a central role in regulating gene expression. Although TF binding to chromatin is primarily dictated by the DNA sequence, the binding patterns can be cell-specific through cooperative interactions between many different TFs. Mutations in cis-regulatory elements can influence TF binding, with potentially deleterious effects [1–3]; therefore, it is important to assess the impact of cis-element mutations on cell-specific TF binding and gene expression. ChIP-seq has been the gold standard for evaluating cell-specific TF binding [4]. However, despite tremendous efforts from the scientific community to generate large-scale TF ChIP-seq data, such as those undertaken by the ENCODE consortium [5], most TFs have been profiled for only a limited number of cells and conditions. Therefore, computational modeling of TF ChIP-seq data to infer the underlying binding rules and to predict TF binding under un-profiled conditions could be a useful and economical approach.

The classical computational model employed to describe TF binding preferences is based on position weight matrices (PWMs) or motifs [6–11]. In addition to binding motifs, other sequence features, such as low-affinity binding sites, flanking DNA, and certain repeat sequence symmetries flanking specific targets, also influence TF binding affinity [12–14]. To better model TF-DNA binding specificity, many DNA-sequence-based models beyond PWM have been proposed [9,13,15,16]. In vivo TF binding is also affected by many other factors such as TF-TF interactions and sequence GC bias. Such information is naturally incorporated in TF ChIP-seq data yet are missing from TF motifs. Therefore, computational models built on ChIP-seq data [17–19] have the potential to capture this information to achieve higher accuracy than those built solely on DNA motifs for in vivo TF binding prediction [20]; however, such studies are limited by the available ChIP-seq data. Last, but not least, the epigenetic landscapes, such as those profiled by histone modification ChIP-seq and DNase-seq, are closely related to TF binding. Computational methods considering motif occurrences in open chromatin regions [21–24] often achieve high accuracy. Such models and TF ChIP-seq data based models may compensate each other.

Deep learning has been actively studied in the machine learning community and has been successfully applied to diverse problems such as image processing, computer vision, speech recognition, and natural language processing over the past decade [25,26]. Recently, deep learning techniques were applied to regulatory genomics and showed exciting performance improvements compared to previous methods [19,27]. Intuitively, when models based on deep learning are trained on ChIP-seq datasets, they can automatically extract sequence features determining TF binding affinity. The extracted features contain not only traditional motifs but also sequence features from a wide window (1,000 bp) flanking the binding sites and TF-TF interactions. However, these models rely on training and cross validation with available ChIP-seq or DNase-seq data and thus are not suitable when such data for a specific cell condition are missing.

In this study, we developed a deep learning method, i.e., TFImpute, to predict cell-specific TF binding based on existing ChIP-seq data. TFImpute embeds cell lines and TFs into continuous vectors that serve as part of the model input. After being trained on available ChIP-seq data from 562 unique TF-cell line combinations (4% of all possible combinations) with 172 transcription factors and 91 cell lines [5], TFImpute predicted TF binding for the remaining 96% TF-cell combinations, for which ChIP-seq data were unavailable. Compared to the existing methods, TFImpute gives more accurate predictions for the binding of TFs on cell types without corresponding ChIP-seq data. Although the imputation of TF binding is considered more challenging than the imputation of epigenetic marks on cell lines without ChIP-seq data [28], our predictions based on TFImpute are highly consistent with results based on massively parallel reporter assays and with evidence in the literature for the selected SNPs.

## Models

**Fig 1:**
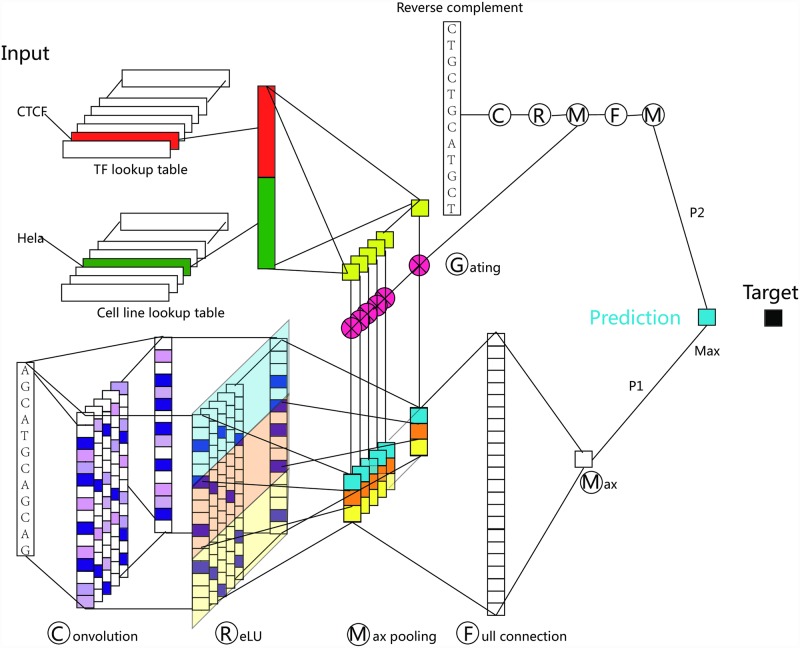
The TFImpute model. Each input is a TF-cell-sequence triple. In the convolution layer, each filter (motif) corresponds to a column. Each filter scans the input sequence and produces one value at each stop. For each filter, the max-pooling layer partitions the signal into three windows and takes the maximum value in each window to obtain three values. The same gate signal operates on the three values, and the gate signal is different for different filters. For each input, the reverse complement of the input sequence together with the TF and cell line is constructed and used as another input for the same network. Therefore, for each input, we obtained two values for forward and reverse strand of the sequence: P1 and P2. The maximum of P1 and P2 is taken as the final prediction. During training, the prediction was compared with the target, and the error was back-propagated to learn the parameters of the whole network.

Given a DNA sequence, a TF and a cell line, TFImpute (Fig 1) predicts whether the TF would bind to the DNA sequence in this cell line. For DNA sequences, the 1-hot encoded signal [19,27] is first passed through a convolution layer [29], which consists of a set of filters. Each of the filters detects whether a specific sequence pattern, i.e. motif, appears in the DNA sequence.

For TFs and cell lines, a technique called embedding [30] is used to encode each TF or cell line as a continuous vector. For each input TF and cell line, their embedding is concatenated and used to calculate a set of gates. The gates are used to tune the signal processed by the convolution layer such that only signals detected by filters related to the current TF and cell line will pass through. For example, if the input DNA sequence contains a motif of the input TF but the TF is not expressed in the input cell line, the signal detected by the filter related to the motif can be tuned down by the gating mechanism.

In the model, embedding for TFs and cell lines, the sequence pattern filters and all other network connections are parameters. To learn these parameters, TFImpute first gives a prediction based on its current parameters and the input, and then compares the prediction with the true label. The difference between the prediction and the truth, i.e. the error, is back-propagated through the whole network, and all the model parameters are updated using AdaDelta [31], a variant of the stochastic gradient descent algorithm (See the Materials and methods section for details).

## Results

### Using random sequences as background

**Fig 2:**
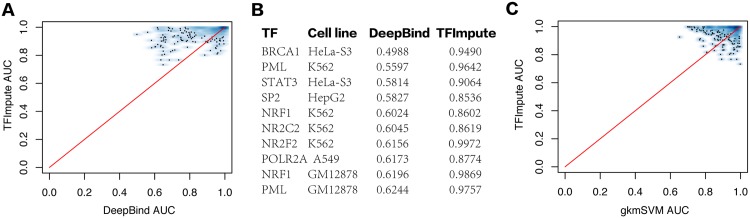
AUC comparison of TFImpute with DeepBind and gkm-SVM using shuffled sequences as negative instances. (A) Comparison with DeepBind. Each point in the figure corresponds to a TF-cell line combination. (B) AUC for TF-cell line combinations in which DeepBind gives the lowest AUC. (C) Comparison with gkm-SVM using randomly shuffled sequences as negative instances. Each point in the figure corresponds to a TF-cell line combination.

To validate the TFImpute model, we first compared it with DeepBind [19], one of the latest deep learning models for TF binding prediction with superior performance to that of several other benchmark methods not based on deep learning. The experimental setting follows [19] with a slight modification. Specifically, we used ChIP-seq peaks from the ENCODE project [5] as the training, validating, and testing data. For each peak region, we utilized 100 bp of DNA sequence around the peak center as the positive peaks and randomly shuffled nucleotides with the number of dinucleotides matching those for each positive peak as the negative peaks. For each ChIP-seq dataset, DeepBind builds a separate model using even-numbered peaks from the top 1,000 peaks as the test set and all other peaks as the training set. For TFImpute, we used the same test set but employed only odd-numbered peaks from the top 1,000 peaks as the training set. In other words, for TFImpute, each ChIP-seq dataset contributed only 500 positive peaks and 500 negative peaks for training. Instead of training a separate model for each ChIP-seq dataset, we trained TFImpute for a single model based on all the ChIP-seq data. The expectation was that the binding information of one TF in one cell line could be helpful for predicting the binding of the same TF or other TFs in other cell lines. For example, the binding sequences of STAT1 and STAT2 should be similar across different cell lines so the training data in one condition may compensate for the lack of training data in another condition. A performance comparison between TFImpute and DeepBind, as shown in Fig 2A and 2B, confirmed the effectiveness of the joint training setting. Although TFImpute uses less training data, it gives better results without the possible drawback of over-training, a serious problem for DeepBind, which requires complicated methods to perform regularization and hyperparameter selection. The underlying reason for the TFImpute to avoid over-training is that TFImpute fits a single model using all the training data but DeepBind fits a separate model for each TF ChIP-seq data set. On the same dataset, we also compared TFImpute with gkm-SVM [15], a state-of-the-art TF binding prediction algorithm not based on deep learning in Fig 2C. Both methods are comparable.

### Using DNase I hypersensitive sites as background

DeepBind defines negative peaks as random sequences in which the number of dinucleotides is matched to the positive set [19]. Another definition of random sequences requires sequence length, GC and repeat fractions that match the positive set [15]. However, in vivo DNA sequences are not random in above senses and the binding sites of TFs are mostly concentrated on active genomic regions, specifically the DNase I hypersensitive sites. Therefore, a set of union DHS sites provide a more biologically meaningful background for TF binding prediction. In this work, our method first defines unified DNase I hypersensitive sites using 122 ENCODE DNase-seq datasets [5] as the background. Then, for each TF ChIP-seq dataset, the cell-specific negative peaks are defined as the unified DNase-seq peak regions that do not overlap with any of the TF peaks in the dataset. Then, the top 5,000 peaks are taken as positive peaks, with each one being matched by a randomly selected negative peak.

We denote the set of TF-cell line combinations as F. To evaluate the performance of TFImpute, we first define 4 subsets (Base, TestSet2, ValidSet2 and TestSet3) of F in Table 1. The specific TF-cell line list for each subset is in S1 Table. Note that the TF-cell line combinations in TestSet3 do not appear in Base where the training set is coming from. In other words, we are training a model using TF binding information in one cell line but verify the model in another cell line. This is biologically meaningful if there are multiple TFs and cell lines involved. For example, if two TFs (TF1 and TF2) were involved and a model learned that TF1 and TF2 are similar due to their similar binding profiles across many cell lines from the training data, it can give a better binding prediction for TF1 in a cell line if the training data contains the binding information of TF2 in this cell line. Therefore, we can evaluate whether a model can utilize such information by training a model for TF1 and TF2 in many cell lines excluding some cell line X and then evaluating the model’s ability to predict TF1 binding in cell line X. The opposite extreme situation is that TF2 is very different from TF1. In this case, the best choice of the model is to ignore TF2 when predicting TF1 binding profile. Although the similarities between TFs or cell lines vary, we hope that the proper designed network structure of TFImpute can learn how to use TF2 in binding prediction of TF1. The designed TestSet3 in Table 1 is to verify whether TFImpute can gain such prediction ability.

**Table 1:** Subsets of TF-cell line combinations using DNase I hypersensitive sites as background.

Definition	Set size	Description
*Base* ⊂ *F*	473	Would be further partitioned to get the training set.
*TestSet*2 ⊂ *Base*	70	Verify the model’s imputation power on replicates.
*ValidSet*2 ⊂ *F* − *Base* − *TestSet*3	20	Monitor over-training for TF-cell line combinations not in the training set. All TF or cell lines in ValidSet2 are thoroughly represented in Base.
*TestSet*3 ⊂ *F* − *Base* − *ValidSet*2	69	Verify the model’s imputation power on TF-cell line combinations not in the training set. All TF or cell lines in TestSet3 are thoroughly represented in Base but do not need to be in ValidSet2.

Each TF-cell line combination corresponds to a set of instances for the input of TFImpute. Each instance is a <1/0, TF, cell line, DNA sequence> quadruple. 1/0 denotes whether the DNA sequence is a TF binding sequence. To simplify the annotation, we also use Base (or TestSet2, ValidSet2, TestSet3) to denote the union of instances corresponding to TF-cell line combinations in Base (or TestSet2, ValidSet2, TestSet3). Instances in Base are further partitioned into 3 disjoint subsets (TrainSet, TestSet1 and ValidSet1) in Table 2.

**Table 2:** Random partition of the instances in Base into 3 disjoint subsets.

Name	#Instances	Description
TrainSet	3,708,593	The training set
ValidSet1	100,000	Monitor over-training for TF-cell line combinations in the training set.
TestSet1	900,000	Verify the model on TF-cell line combinations in the training set.

During calculation, we first maximize the set size of the union of ValideSet2 and TestSet3. Then randomly selected 20 of them as the ValidSet2. Finally, we maximize the set size of TestSet2. The specific TF-cell line list for each subset is in S1 Table. Because ChIP-seq experiments are not evenly distributed for TFs or cell lines, subsets defined above are biased towards TFs and cell lines that have many ChIP-seq experiments in the dataset. This is a possible mechanism of overfitting.

### Using GC matched negative instances

For a comprehensive comparison with gkm-SVM, we also applied TFImpute to the dataset in [15], where the GC matched sequences are used as negative instances. Similar to Tables 1 and 2, we partitioned the dataset into 6 subsets: TrainSet, ValidSet1, TestSet1, ValidSet2, TestSet2, and TestSet3. The basic statistics for each subset is in Tables 3 and 4, S2 Table

**Table 3:** Subsets of TF-cell line combinations using GC matched negative instances.

Definition	Set size
*Base*	339
*TestSet*2	67
*ValidSet*2	20
*TestSet*3	41

**Table 4:** Random partition of the instances in Base of Table 3 into 3 disjoint subsets.

Name	#Instances
TrainSet	2000000
ValidSet1	200000
TestSet1	696076

### Comparisons with gkm-SVM and PIQ

To compare TFImpute with gkm-SVM [15] and PIQ [22], we apply the default parameters for both gkm-SVM and PIQ. gkm-SVM cannot accept inputs from multiple conditions; thus, we trained gkm-SVM separately on each TF-cell line combination on the union of the training and validation sets. PIQ does not need a training set but accepts a genome-wide DNase I hypersensitivity profile and a known motif to predict the binding of a TF. We apply PIQ on TestSet3 with matched cell line DNase I hypersensitivity profiles. The required motifs for running PIQ could be found in S3 Table. (See Material and methods for more details).

**Fig 3:**
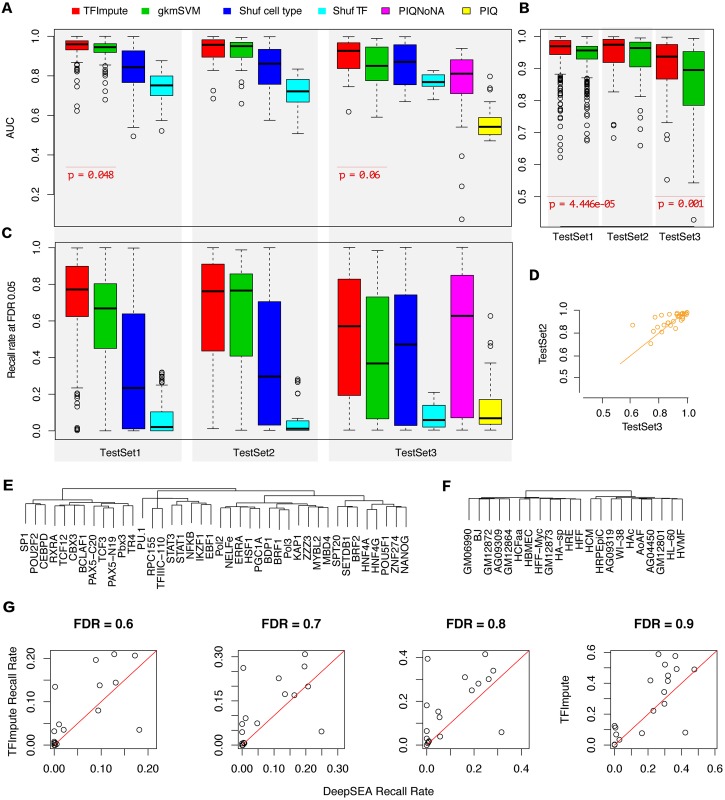
Comparison with gkm-SVM, PIQ, and DeepSEA. (A) AUC comparison of TFImpute and gkm-SVM on TestSet1, TestSet2, and TestSet3. ‘Shuf cell line indicates that the cell line of the corresponding test set was shuffled and that the trained TFImpute model was then applied to the shuffled dataset. Similarly, ‘Shuf TF’ indicates that the TFs were shuffled. For some of the given regions, PIQ give NA predictions. NA means that there is no motif based on log probability threshold of 5, or the region is lack of DNase I signal. PIQNoNA in this figure denotes the result after removing all NAs and PIQ denotes the result after treating NAs as no binding. To calculate the AUC, the predictions were grouped by TFs. The middle bar in each box indicates the median. (B) AUC comparison based on predictions grouped by TF-cell line combinations. (C) The recall rates of different methods at FDR 0.05 (See Material and methods for more details). The predictions were grouped by TFs. (D) AUC comparison of TFImpute on TFs appearing in both TestSet2 and TestSet3. (E) Hierarchical clustering of a subset of the TFs based on the learned embedding by TFImpute. The full clustering is shown in S3 Fig. (F) Hierarchical clustering of a subset of cell lines based on the learned embedding by TFImpute. The full clustering is shown in S4 Fig. (G) The recall rate of TFImpute and DeepSEA at different FDR cutoffs on the datasets provided by DeepSEA.

A comparison is shown in Fig 3A. For TestSet1, TFImpute achieved an average AUC above 0.94 and performed slightly better than gkm-SVM. For TestSet2, the set to verify the accuracy of the methods on ChIP-seq replicates, both methods were comparable with no statistically significant difference. The most important result was observed for TestSet3, in which the tested TF-cell line combinations were not in the training set. To evaluate the performance of gkm-SVM on TestSet3, we randomly selected a trained gkm-SVM model based on TrainSet with matched TFs for each dataset in TestSet3. For TestSet3, TFImpute performed much better (average AUC 0.895) than gkm-SVM (average AUC 0.852). The p-values (0.048 for TestSet1 and 0.06 for TestSet3) between TFImpute and gkm-SVM AUC indicate that the differences are not very significant. The main reason for this finding is that the numbers of data points are small. When the predictions were grouped by TF-cell line combinations, we obtained more significant p-values (4.446e-05 for TestSet1 and 0.001 for TestSet3; Fig 3B). To rule out the possibility that TFImpute outperforms gkm-SVM by chance, we randomly partition Base 5 extra times based on Table 2 and check the variability of TFImpute AUC (S1A Fig). The results show that the variation of TFImpute AUC is very small. On TestSet3, PIQ achieves the lowest AUC. A possible reason is that PIQ does not give predictions on regions without strong motif or regions lack of DNase I signal. Therefore, we removed such regions before calculating the AUC (PIQNoNA in Fig 3A). We found that TFImpute still outperforms PIQ in this case.

Because a TF binds to only a small proportion of the chromatin accessible regions, methods that can impute TF binding with low FDR are more valuable. In Fig 3C, we compared the recall rates of different methods at fixed FDR 0.05. On TestSet1 and TestSet3, TFImpute outperforms gkm-SVM and PIQ. The conclusion also holds at FDR 0.01 and FDR 0.1 (S1B Fig). Another important application of TF binding prediction is to scan possible binding sites genome-widely. We evaluated the performances of the three methods on non-overlapping bins of length 300bp on chr8. Then we applied the trained models of TFImpute, gkmSVM on TrainSet to TF and cell line combinations in TestSet3 on chr8, and extracted the maximum score of PIQ calls for each bin on chr8. Note that PIQ needs associated DNase-seq data for each cell line but TFImpute and gkmSVM are solely based on DNA sequence. The comparisons shown in S1C Fig demonstrate that all three methods give high false positive rates, which is consistent with the literature [21,32,33]. TFImpute and gkmSVM are comparable and relatively better than PIQ at large FDR cutoffs (95% and 99%). At FDR cutoff 50%, PIQ achieves higher recall rates. The low recall rate of TFImpute is partially due to the fact DNase region instead of the whole genome were used as the background during training. The following comparison with DeepSEA shows that the recall rate increases as wider regions were used as background.

To further confirm the super performance of TFImpute over gkm-SVM, we further applied both methods on the dataset used to validate gkm-SVM [15]. This dataset uses GC matched negative regions. The AUC and recall rate comparison demonstrate that TFImpute outperforms gkm-SVM consistently (S2 Fig).

We also compared the running time of TFImpute, gkm-SVM and PIQ in Table 5. The total time costs of three methods are comparable. We didn’t test TFImpute on CPU because CNN neural networks are generally extremely slow on CPU. TFImpute doesn’t have running time for each dataset separately because all the training data are supplied to TFImpute jointly. The running time of gkm-SVM and PIQ is the average time of processing all the datasets.

**Table 5:** Running time of different methods.

	Hardware	Training Time
TFImpute	GPU GTX970	80h/all dataset
gkm-SVM	CPU AMD Phenom(tm) II X4 910e	38min/dataset
PIQ	CPU AMD Phenom(tm) II X4 910e	40min/dataset

Finally, we compared the performance of TFImpute on TestSet2 and TestSet3. Because the TFs in TestSet3 were different from those in TestSet2, the differing performance of TFImpute on the two test sets may not be directly comparable with the overall AUC shown in Fig 3A. Therefore, we focused on transcription factors that appeared in both TestSet2 and TestSet3. For most of these TFs, TFImpute performed equally well (Fig 3D), indicating that the learned interactions of TFs and cell lines determined by TFImpute can be generalized to novel TF-cell line combinations. This benefit is highly valuable because most of the TF-cell line combinations don’t have corresponding ChIP-seq data.

### Comparisons with DeepSEA

Because the released DeepBind software package does not contain the training step, we could not train it on our dataset. Therefore, we compared TFImpute with DeepSEA on the datasets provided in [27]. This dataset defines the background as regions where at least one TF binds in at least one of the cell lines. Such a wide background gives us the opportunity to estimate the recall rate of a method on certain FDR cutoffs genome-widely.

To compare with DeepSEA genome-widely, we transformed the data from DeepSEA paper into the format required by TFImpute. We faced three obstacles. First, the input of TFImpute requires <DNA sequence, TF, cell line > triples. If we use all negative peaks in the data provided by DeepSEA to train TFImpute, the data would be too large to be trained. Second, given a <DNA sequence, TF, cell line> triple, the current TFImpute model predicts whether the TF would bind to the DNA sequence in the cell line. It is too inefficient to cases if we want to predict the binding of a set of TFs on a DNA sequence in a cell line. Third, the input DNA sequence of DeepSEA is 1000bp and the DeepSEA model used three layers of convolutions to handle the long DNA sequence. The TFImpute model was designed for relatively short sequences and therefore only one layer of convolution was used to save computation time.

We found no easy way to solve above three problems simultaneously. The main reason is that the TF and cell line information have to be input to the model in some way to keep the imputation ability. This imputation ability dramatically increases the required computation. Therefore, we conducted the following procedures to partially solve the above problems and compared TFImpute with DeepSEA genome-widely.

First, to train TFImpute we excluded histone modifications and DNase-seq data and sampled one negative peak for each positive peak to reduce the data size. For DeepSEA, we used full dataset and reproduced the prediction results in the DeepSEA paper. Second, to compare with DeepSEA genome-widely, we queried the Cistrome Data Browser database [34] for another 19 TF ChIP-seq datasets (S4 Table) not included in the training set (See Material and methods for more details). In the 19 datasets, the TF and cell line appears in the training set but the TF-cell line combinations are not contained in the training set. Then we partitioned chr8 into non-overlapping bins of length 1000bp and evaluated TFImpute and DeepSEA on chr8. For each ChIP-seq dataset, a bin is considered as having TF binding if there is a peak center in the bin. Because the TF-cell line combinations of the 19 datasets are not contained in the training set, DeepSEA cannot give predictions for the right cell line for each TF. Therefore, we selected the prediction of a random cell line in the training set for each TF to form the final prediction of DeepSEA. Third, we adjusted the network structure of TFImpute such that it can handle 1000bp DNA sequence without imposing extra computation needs and can predict the binding of a set of TFs on a DNA sequence in a cell line simultaneously (See Material and methods for more details).

We compared the recall rates of DeepSEA and TFImpute at different FDR cutoffs. Generally, the recall rates at low FDR are quite low for both methods. To get a reasonable recall rate (e.g. 20%), the FDR should be above 50%. The recall rates at 4 FDR cutoffs (60%, 70%, 80% and 90%) are shown in Fig 3G. The TFImpute model outperforms DeepSEA for majority of the cases.

### Sequence features are the primary factors influencing TF binding

The TFImpute model provides the ability to estimate whether sequence features or cell lines have a more significant influence on TF binding. Fig 3A and 3C show that if a TF does not match the DNA sequences and cell lines (by shuffling TFs in the test set), the prediction accuracy drops more statistically significantly than when cell lines are shuffled. This result indicates that sequence features are the dominant factor in determining TF binding compared to cell line. Because randomly selecting a trained model based on TrainSet with matched TFs for each dataset in TestSet3 is equivalent to ignoring cell- specificity, we observed that the performance of TFImpute with shuffled cell lines was comparable to that of gkm-SVM on TestSet3 (Fig 3A).

Because each TF or cell line had learned embedding, we could compare the similarities between embedded vectors. We observed that many TFs with similar known motifs were grouped together, such as HNF4G/HNF4A and STAT1/STAT3 (Fig 3E, S3 Fig). The meaning of embedding for cell lines is subtler because it is not the primary factor influencing TF binding. Therefore, we did not observe clear patterns in clustering for cell lines (Fig 3F, S4 Fig).

### Enhancer activity is correlated with the binding prediction based on TFImpute

**Fig 4:**
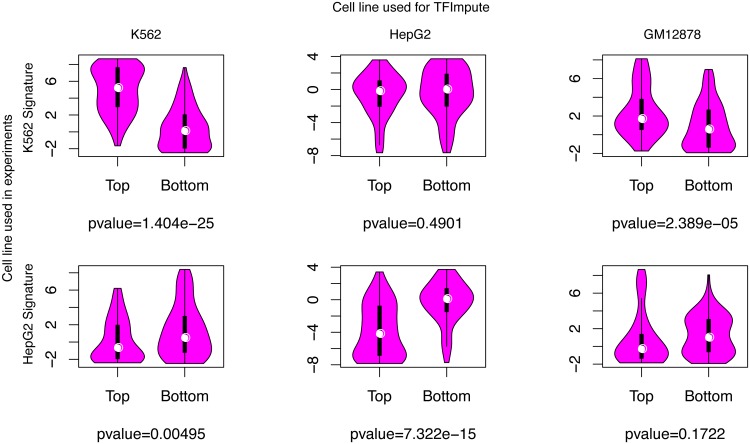
The distributions of the calculated enhancer signature for the top and bottom 100 enhancers. The p value is calculated using t-test. We would like to emphasize the lack of data of the enhancer reporter assay of GM12878, which is a good control.

To systematically estimate the efficacy of TFImpute, we applied TFImpute on a massively parallel reporter assay that experimentally characterized the role of enhancer sequences by introducing target mutations in K562 and HepG2 cell lines [35]. For a specific cell line and each of the enhancer sequences, TFImpute gave TF binding predictions for all the TFs in the training set. To obtain a summarized enhancer activity prediction for each enhancer sequence, we performed principle component analysis (PCA) on the matrix of the predicted binding affinity for all TFs and enhancer sequences. The first principle component was taken as a signature of enhancer activity for each sequence. Then, we compared the signature value distribution of the top 100 enhancer sequences from that of the bottom 100 enhancer sequences after all the enhancer sequences were ranked by their experimentally measured activities. Fig 4 shows that the difference is much more statistically significant if the cell lines are matched. The same relationship is also confirmed by the Spearman rank correlation analysis shown in S5 Fig. When the cell lines are matched, the p values for the Spearman rank correlation are much more statistically significant than other cases. In S5 Fig, it is interesting to note that the correlation coefficient is positive (0.5747) in K562 but negative (−0.4422) in HepG2. This is reasonable because we would expect that the set of TFs binding to the active enhancers of K562 is different from that of HepG2. It also means that the signature based on TF binding prediction should not be considered as a prediction of the enhancer activity.

### TFImpute accurately predicts the impact of SNPs on TF binding

**Fig 5:**
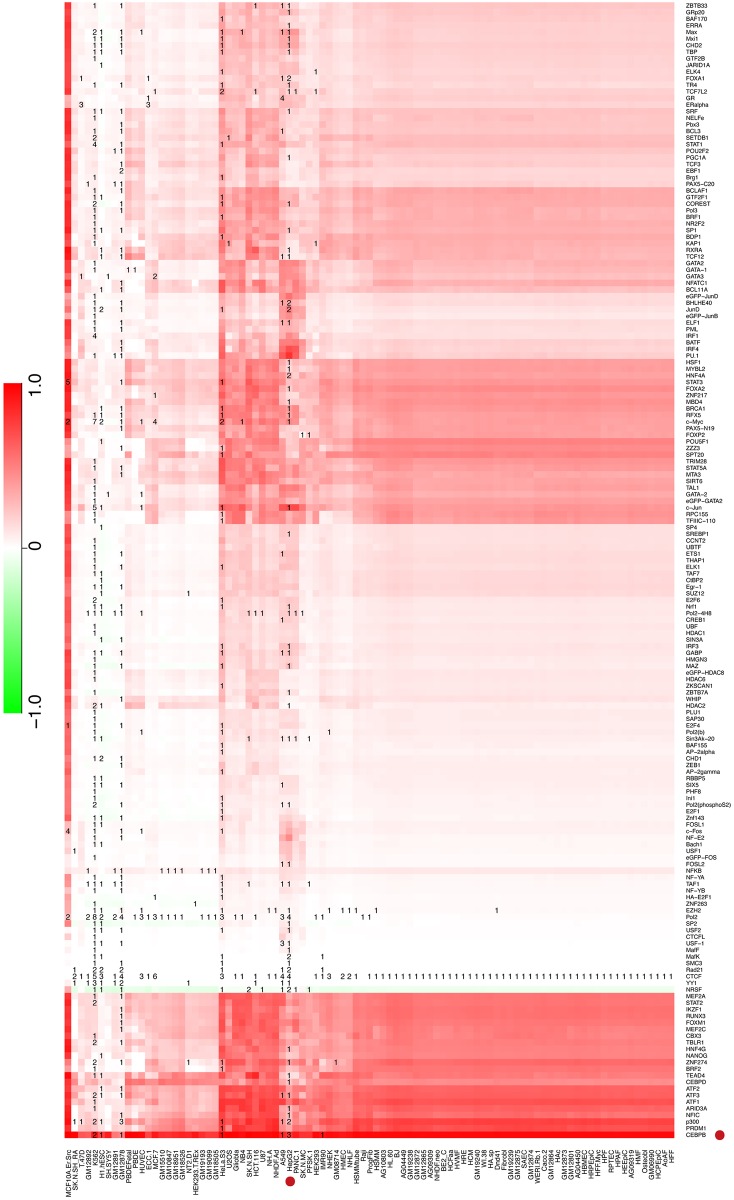
Predicted binding affinity change between two alleles of SNP rs12740374 (T/G). The color in each cell represents the predicted binding affinity of allele G minus that of allele T for the corresponding TF and cell line. The number in each cell of the heatmap is the number of ChIP-seq datasets in the training set for the corresponding TF and cell line. If TFImpute predicted strong binding in the minor allele but no binding in the major allele, the score was 1. If TFImpute predicted no binding difference between the two alleles, the score was 0.

Because TFImpute can accurately predict TF binding, we can use the trained TFImpute model to estimate TF binding differences in the minor and major alleles of individual SNPs for all possible TF-cell line combinations if the TF and the cell line appear in the training set. To validate the accuracy of TFImpute on mutated sequences, we filtered 10 SNPs from the literature that were reported to lead to differential binding for TFs and cell lines appearing in our training set. TFImpute gave predictions consistent with the literature for 7 of these SNPs (S5 Table). For example, in liver, the minor allele (T) of SNP rs12740374 created a binding site for C/EBP, and the major allele (G) disrupted the binding [24]. TFImpute accurately predicted that the C/EBP binding affinity would increase when the DNA sequence was changed from the major allele to the minor allele in HepG2, a liver hepatocellular carcinoma cell line (Fig 5). As another example, in SNP rs4784227 (C/T), a functional variant in breast cancer, the risk-associated allele (T) increases FoxA1 binding in breast cancer [36]. Although there is no ChIP-seq data for FoxA1 in MCF-7 in the training set, TFImpute predicted that the binding of FoxA1 would increase if the input sequence at the SNP location were changed from C to T in MCF-7 (S6 Fig). Moreover, for SNP rs4953223 (C/T), one of the DNase I—sensitivity quantitative trait loci (dsQTLs) in human lymphoblastoid cell lines (LCLs) [37], the T allele disrupts the binding of NF-kB and is strongly associated with low DNase I sensitivity [24]. TFImpute accurately predicted that the binding affinity of NF-kB would dramatically decrease in most of the cell lines, including all the GM cell lines (lymphoblastoid cell lines), if the DNA sequence at rs4953223 was changed from C to T (S7 Fig). Moreover, dozens of other TFs, such as EBF1 and STAT1, also showed clear binding affinity decreases in most of the GM cell lines.

## Discussion

The most efficient resource for studying TF binding is ChIP-seq. However, a ChIP-seq experiment is limited by its cost, the availability of proper antibodies, and the complexity of the protocol. Therefore, only a small fraction of possible ChIP-seq experiments have been performed, considering all possible TF-cell line combinations. Because TF binding is a cell-specific behavior, it is not optimal to transfer a trained TF binding prediction model without condition-specific features, such as sequence-only models of TF binding, from one condition to another. TFImpute provides an economical method for alleviating this problem. Compared with existing methods, the properly formed multi-tasking learning framework along with the embedding and the gating mechanism make TFImpute obtain more accurate predictions for TF-cell line combinations without ChIP-seq data, given that the TF and the cell line appear in the training set. As more ChIP-seq data are used to train the model, TFImpute will be more powerful because the number of TF-cell line combinations increases quadratically with the number of TFs and cell lines.

To summarize enhancer activities, we utilized the first PCA component of the predicted TF binding score for all TFs and enhancer sequences. This method represents a rather simplified approach. It is expected that the use of more PCA components would provide a better fit to the enhancer activity, which was observed. However, even when all the components were used to fit the activity of all enhancers using linear regression, we did not obtain a perfect fitting (r-squared are 0.1361 and 0.2359 for K562 and HepG2 respectively), for at least two underlying reasons. First, the measured enhancer activity was noisy [35]. Second, the activity of the enhancers was not linearly dependent on the TF binding signal. To address the non-linearity, a more dedicated model might be needed.

The trained TFImpute model based on non-mutated sequences is well suited for predicting the binding of TFs on mutated sequences such as SNPs. Similar results have also been observed in previous studies, such as [24,27]. Such models are valuable for studying the gene regulatory network *in silico*, which is more rapid and economical than wet lab experiments. The unique imputation ability of TFImpute further extends the predictive power to some TF-cell line combinations without corresponding ChIP-seq data. Such an extension complements models based on sequence features and open chromatin profiling data, such as PIQ [22].

It is worth noting that the prediction reliabilities are different for different TF-cell line combinations without ChIP-seq data. Fig 5 clearly demonstrates this phenomenon. For approximately one half of the cell lines, only CTCF ChIP-seq data are available; therefore, the binding predictions are very similar for these cell lines. These predictions cannot intuitively reflect the cell-specificity. Important future work should include the quantitative estimation of the prediction reliability.

## Materials and methods

### The deep learning neural network

A deep learning neural network transforms inputs through multiple layers of simple computation units. Each unit provides very limited computations such as performing a summation or calculating the sigmoid function. Normally, computation units are connected only to adjacent layers. Similar to [19,27], our TFImpute model is also based on a convolutional neural network (CNN) [29]. However, our approach differs in that we also use TFs and cell lines as part of the input with embedding. Embedding is a technique that allows discrete features to be represented with continuous vectors. This technique has wide applications in natural language processing to represent words or relations [30]. With the embedding of TFs and cell lines, we can train the model to predict whether a sequence will exhibit cell-specific TF binding, even for TF-cell line combinations without data for all TFs and cell lines in the training set.

The 1-hot encoding method [19,27] represents each DNA sequence of length N by an N*4 matrix. Each column of the matrix corresponds to A, C, G, or T, and each row corresponds to a sequence location. For the i^th^ row of the matrix, we set the 1st (2nd, 3rd, or 4th) column to be 1 if the i^th^ j^th^ element of the sequence is A (C, G, or T), and all remaining elements of the matrix are set to 0.

A convolution layer first transforms the DNA sequence with multiple filters. Each filter can be conceptually considered as a motif. Specifically, the output of the convolution layer with input matrix *X* is another matrix *Y* of size (N-F+1) * K, where *F* is the length of the filter and *K* is the total number of filters. *Y* is defined as

$$Y_{r,k}=\overset{F}{\underset{i=1}{\sum }}\overset{4}{\underset{j=1}{\sum }}W_{i,j}^{\left(k\right)}X_{r+i-1,j}$$

where *W*^(*k*)^ is a parameter matrix for the *k*^*th*^ filter and r is the filter position.

Next, a ReLU layer operates on the input matrix element by element. This layer filters out noise and keeps only significant signals by

$$\text{ReLU}\left(x,b\right)=\{\begin{array}{c} x\;\;\;\;if\;(x>b)\\ 0\;\;\;otherwise \end{array}$$

where *b* is a parameter to reflect the fact that different motifs may have different binding affinities.

Then, a max-pooling layer follows. This layer takes the maximum value of the signal on non-overlapping windows. Intuitively, this layer detects whether a motif occurs in a window while ignoring the exact location of the occurrence.

After the pooling layer, the network goes through a gating layer. The gates are determined by the input TF and cell line. The purpose of gating is to further tune the signal such that only signals from motifs related to the current input TF and cell line pass through.

The calculation of gates is based on the embedding for the input TF and cell line, each of which is associated with a 1-dimensional continuous vector with 50 float elements. Given the input, the model first goes through a lookup table to obtain the embedding for the input TF and cell line. Then, a concatenated embedding vector goes through a one-layer neural network with sigmoid activations. In this way, the neural network can model complex interactions between TFs and cell lines to determine whether a TF will bind to a specific sequence in a specific cell line. This gating strategy differs from the method used by [38], which simply supplies the cell line or TF using a 1-of-*N* coding as an input to the network. The complete formula for describing the model is as follows:

$$G\left(X\right)=sigmoid\left(W_{1}embed\left(X,\;E\right)+b_{1}\right)$$

M(S, X)=G(X)*Pooling(ReLU(conv(S,W),b))

$$F\left(S,X\right)=\;\text{max}(W_{2}M\left(S,X\right)+b_{2})$$

Y=max(F(S,X),F(revcomp(S,X)))

where *X* is the input TF and cell line; E is the embedding matrix for all TFs and cell lines; embed(X, E) simply concatenates the embedding vectors for the TF and the cell line from X; and *S* is the input sequence. *revcomp* takes the reverse complement of the input sequence, and E,W_1_, W_2_, b_1_, b_2_, b and W = {W^(k)^, 1 ≤ k ≤ F} are model parameters. Because the embedding for either the TF or the cell line is a 50-dimension vector, the output of embed(X, E) is a 100-dimension vector. Once the model has been trained, the embeddings for TFs and cell lines in the training set will be learned. For TFs or cell lines not in the training set, the model cannot be applied.

TFImpute achieves cell line specificity in a very different way from that of DeepSEA [27]. In that study, all the predictions shared the whole network, except for the last two layers. Therefore, the ability to differentiate various TFs and cell lines was limited by the model ability of the last two layers. Moreover, DeepSEA can model only TF-cell line combinations that appear in the training data and cannot extrapolate to cases for further TF-cell line combinations.

### The revised neural network structure for comparison with DeepSEA

To compare with DeepSEA on long DNA sequence, we revised the network structure of TFImpute as follows. First, each cell line is associated with an embedding with length equal to the number of motifs (2000). Then a sigmoid function is applied on the embedding to get the gates. Second, F(S, X) is replaced by taking maximum over non-overlapping window with size 50 followed by GRU [39], a recurrent layer, with hidden size 1000. The hidden state of the GRU at the last time step is connected to the output. Third, the number of outputs is equal to the number of TFs in the training set. In other words, the revised model is a CNN-RNN structure with cell line embedding as gates. Such a model keeps the imputation ability, gives predictions for multiple TFs in a cell line simultaneously and handles long DNA sequences without imposing extra computation needs. During training, the learning rate decay was 0.01 for every 200,000 instances and the best model is selected based on valid set.

### Data source for generating datasets

Thanks the authors of [40], we get a copy of the processed union of DHS. Briefly speaking, this union DHS are constructed based on the 122 ENCODE [5] DNaseI-seq data. Each DNase peak was trimmed to 300 bp centered at its summit. Then, the peaks are merged to get the union DHS regions. The final DHS region contains 409303942 bps, approximately 13% of the genome. We downloaded 690 TF ChIP-seq datasets of the ENCODE project [5] from http://hgdownload.cse.ucsc.edu/goldenPath/hg19/encodeDCC/wgEncodeAwgTfbsUniform/. This dataset contained 562 unique TF-cell combinations, with 172 TFs and 91 cell lines. For datasets with GC matched negative instances [15], the data has been downloaded from http://www.beerlab.org/gkmsvm/downloads/sequences_gkmsvm.tar.gz.

### Model training for TFImpute

To train the model based on random sequences, the odd-numbered peaks in the top 1,000 peaks for each dataset were randomly partitioned into a training set (280,000 instances) and a validation set (31,102). The neural network was trained by scanning the training set over 50 epochs, and the performance on the validation set after each epoch was recorded. Finally, the best-performing model was applied to the test set.

To train the model based on DNase I hypersensitive sites, the performance of the model was evaluated on ValidSet1 and ValidSet2 for every 50,000 instances. Then, the best-performing models over 10 epochs were taken as the final models. The best model based on ValidSet1 was used on TestSet1 and TestSet2, and the best model based on ValidSet2 was used on TestSet3. When applying TFImpute to estimate the effect of SNPs on TF binding affinity and to estimate the enhancer activity, TrainSet, TestSet1, ValidSet1, TestSet2, and TestSet3 were merged and used for training, and ValidSet2 was used for validation to select the optimal early stop point. Then, the stop point and all the data including ValidSet2 were used to train the final model. The model was trained on a GPU (GTX970) for 80 hours to finish 10 epochs.

### Hyperparameters for TFImpute

For comparison with DeepBind, the input sequence length was 100. The motif length was set to 20, and the number of motifs was set to 600. The window size for the max-pooling layer was 100; thus, only one maximum value was calculated for each motif. The full connection layer size was set to 50. The learning rate decay was 0.03 for each epoch. For the model based on DNase I background, the input sequence length was 300. The motif length was set to 20, and the number of motifs was set to 2,000. The window size for the max-pooling layer was 100; thus, three maximum values were calculated for each motif. For each situation, all the weights were randomly initialized with a Gaussian distribution of mean 0 and standard deviation 0.01. The bias *b* for the rectification layer was initialized to -4. All other biases were initialized to 0. The learning rate decay was 0.03 for every 50,000 instances.

### Procedures of applying PIQ

We compared TFimpute with the latest version of PIQ (http://bitbucket.org/thashim/piq-single/src). We consulted the author of PIQ for the parameters and usage of PIQ. The procedure is as follows. First, the DNase-seq data was obtained from http://hgdownload.cse.ucsc.edu/goldenPath/hg19/encodeDCC/wgEncodeUwDgf, http://hgdownload.cse.ucsc.edu/goldenPath/hg19/encodeDCC/wgEncodeOpenChromDnase, and http://hgdownload.cse.ucsc.edu/goldenPath/hg19/encodeDCC/wgEncodeUwDnase. Replicate DNase-seq data from the same ENCODE participant lab were merged. For the DNase-seq experiments in the same cell line performed by different labs, the one with the maximum number of mappable reads was selected. Second, we used the motif reference table from PIQ (http://piq.csail.mit.edu/data/misc/integration.test.csv) to match the transcription factor name with PWM id in JASPAR database. For transcription factor names without PWM annotations, we combined the PIQ transcription factor calls from http://piq.csail.mit.edu/data/141105-3618f89-hg19k562.calls/141105-3618f89-hg19k562.calls.tar.gz and ENCODE ChIP-seq peaks to follow the routine suggested by the PIQ author to get the most predictive PWM of the ChIP-seq peaks as in http://piq.csail.mit.edu/data/misc/compare.tar.gz. Last, we used the default parameters to run PIQ for the 69 <TF, PWM, DNase-seq, cell line > combinations (S3 Table) whose TF-cell line combinations are in TestSet3 (S1 Table), and got the maximum purity score of the positive and negative strand for each region. For genome wide transcription factor binding prediction, we run the same procedure as above, but replaced the background input region with the 300bp windows on chr8 for the TF-cell combinations in TestSet3.

### External dataset from Cistrome Data Browser

We queried the Cistrome Data Browser database [34] for human ChIP-seq datasets satisfying the following two conditions: First, the TF and the cell line combinations are not in the TF ChIP-seq datasets from DeepSEA [27], but the TF or the cell line separately should be in the datasets. In this step, both the official name and its aliases for a TF are considered. Second, the number of peaks with good quality (Q-value < = 0.01) is larger than 20,000. The resulted datasets were manually selected to make the TF and cell line name as unique as possible. Finally, 19 ChIP-seq datasets remain (S4 Table).

### The calculation of recall rate at certain FDR cutoff

We calculated the threshold for the predicted score based on the test data for a certain FDR cutoff. Then, the recall rate is calculated based on the threshold. We didn’t calculate the FDR using training data to make the metric definition consistent for all methods compared in this work including PIQ that does not need a separate training set.

### TFImpute package

The TFImpute method was implemented using Theano [41,42]. The software is available at https://bitbucket.org/feeldead/tfimpute.

## Supporting information

S1 Table The specific TF-cell line list for each subset of Table 1. (CSV)

S2 Table The specific TF-cell line list for each subset of Table 3. (CSV)

S3 Table The motif IDs for running PIQ on TestSet3. (TXT)

S4 Table Selected 19 TF ChIP-seq datasets from Cistrome Data Browser database [34]. (TXT)

S5 Table Predicted binding differences for the major and minor alleles of SNPs. (XLSX)

S1 Fig (A) The AUC of TFImpute at different random partitions of Base based on Table 2. Run0 corresponds to the result in Fig 3A. (B) The recall rates of TFImpute, gkm-SVM and PIQ at FDR 0.01 and 0.1 on union DHS regions. (C) The recall rates of the three methods at FDR 0.5, 0.9, 0.95, 0.99.
(TIFF)

S2 Fig AUC and recall rate comparison of TFImpute and gkm-SVM on datasets using GC matched negative instances. The predictions were grouped by TFs.
(TIFF)

S3 Fig Hierarchical clustering of TFs based on their learned embedding. (TIF)

S4 Fig Hierarchical clustering of cell lines based on their learned embedding. (TIFF)

S5 Fig Spearman rank correlation between the enhancer sequence and the predicted value. (TIFF)

S6 Fig Predicted binding affinity change between two alleles of SNP rs4784227 (C/T). The color in each cell represents the predicted binding affinity of allele T minus that of allele C for the corresponding TF and cell line.
(TIFF)

S7 Fig Predicted binding affinity change between two alleles of SNP rs4953223 (C/T). The color in each cell represents the predicted binding affinity of allele T minus that of allele C for the corresponding TF and cell line.
(TIFF)
