# Supplementary figures and images for: Imputation for transcription factor binding predictions based on deep learning
Source: PLoS Comput Biol. 2017 Feb 24;13(2):e1005403. doi: 10.1371/journal.pcbi.1005403 (PMC5345877; doi:10.1371/journal.pcbi.1005403)

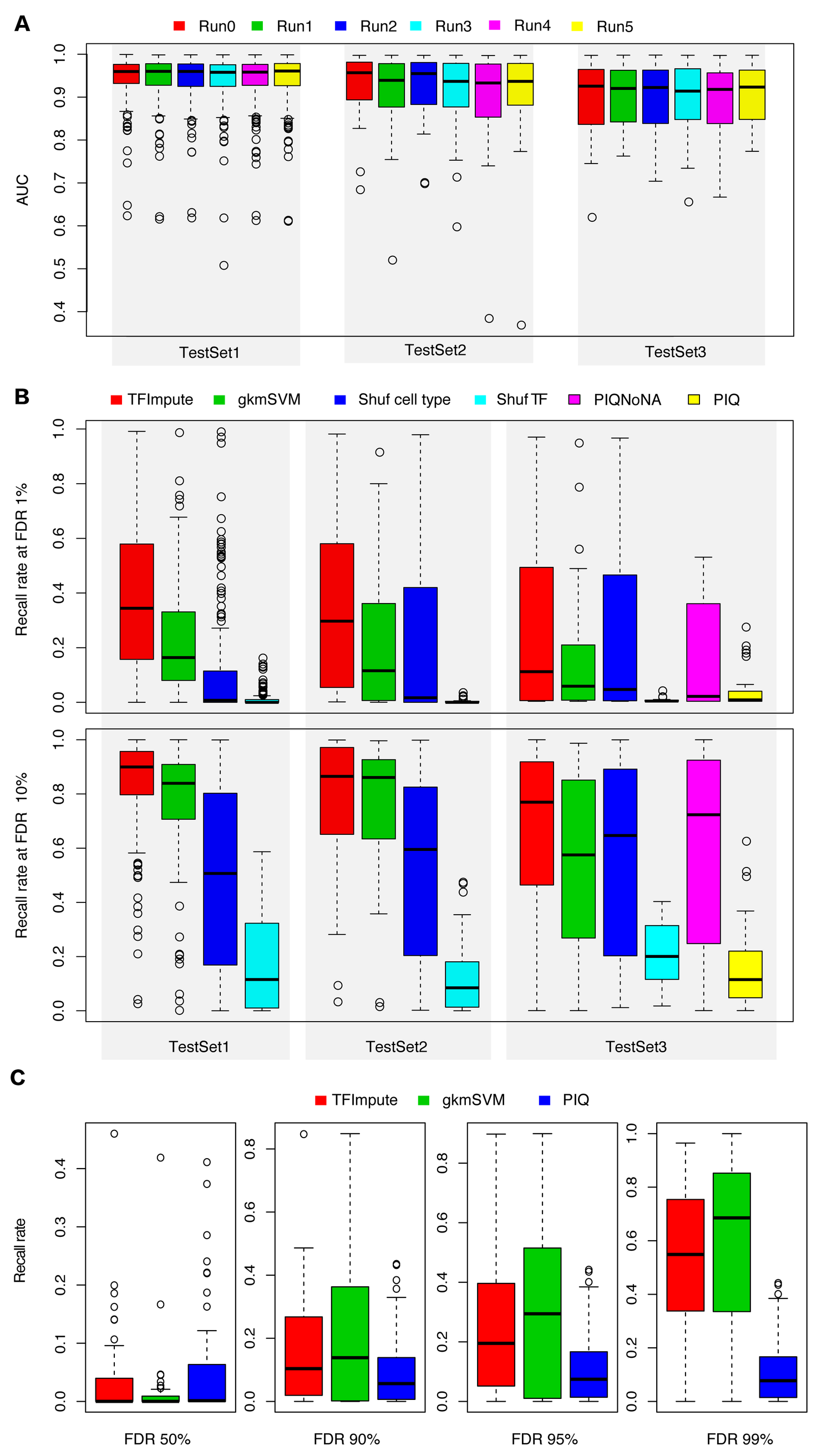

Supplement: S1 Fig — (A) The AUC of TFImpute at different random partitions of Base based on Table 2. Run0 corresponds to the result in Fig 3A. (B) The recall rates of TFImpute, gkm-SVM and PIQ at FDR 0.01 and 0.1 on union DHS regions. (C) The recall rates of the three methods at FDR 0.5, 0.9, 0.95, 0.99. (TIFF) [file pcbi.1005403.s006.tiff]

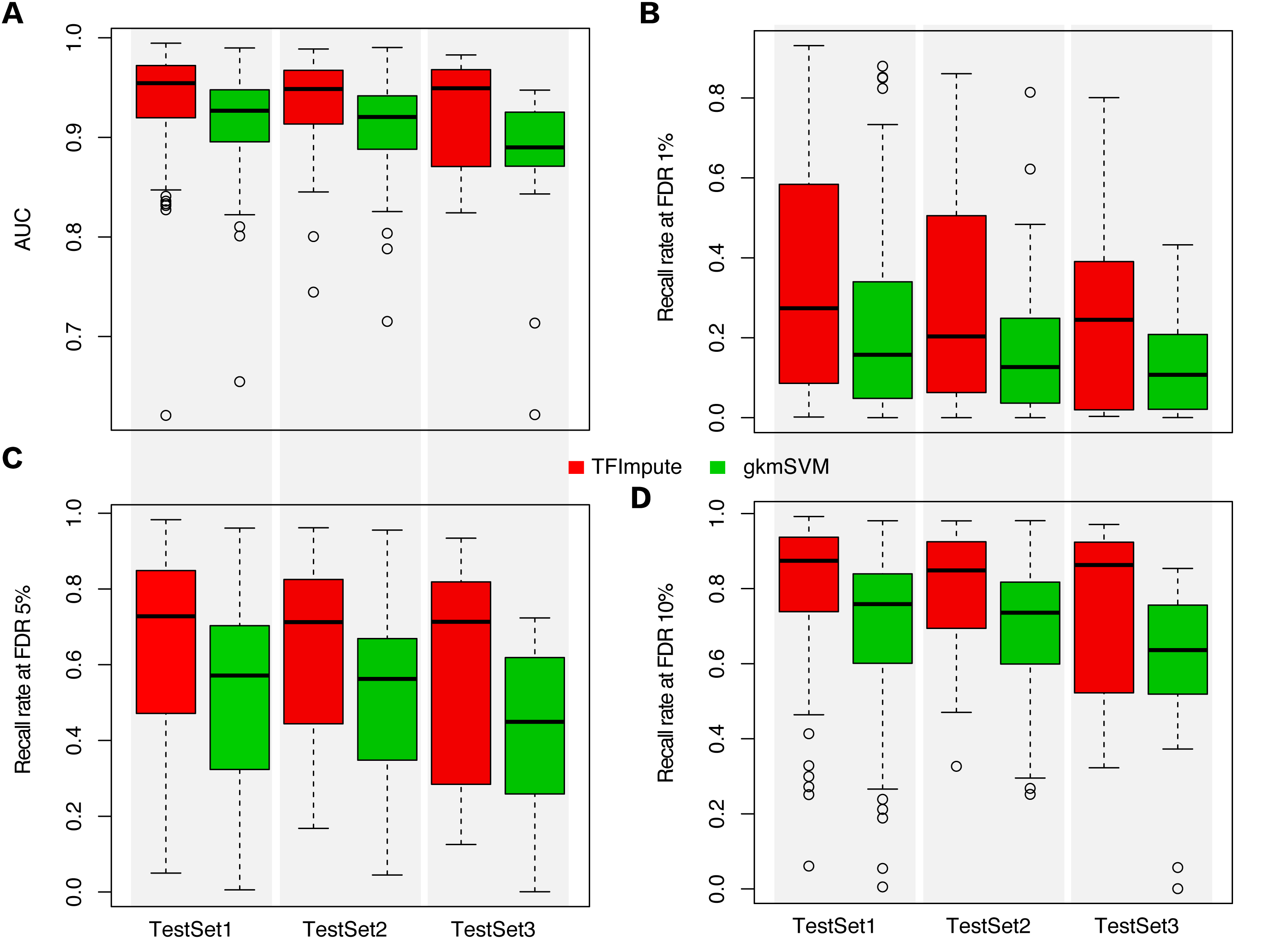

Supplement: S2 Fig — The predictions were grouped by TFs. (TIFF) [file pcbi.1005403.s007.tiff]

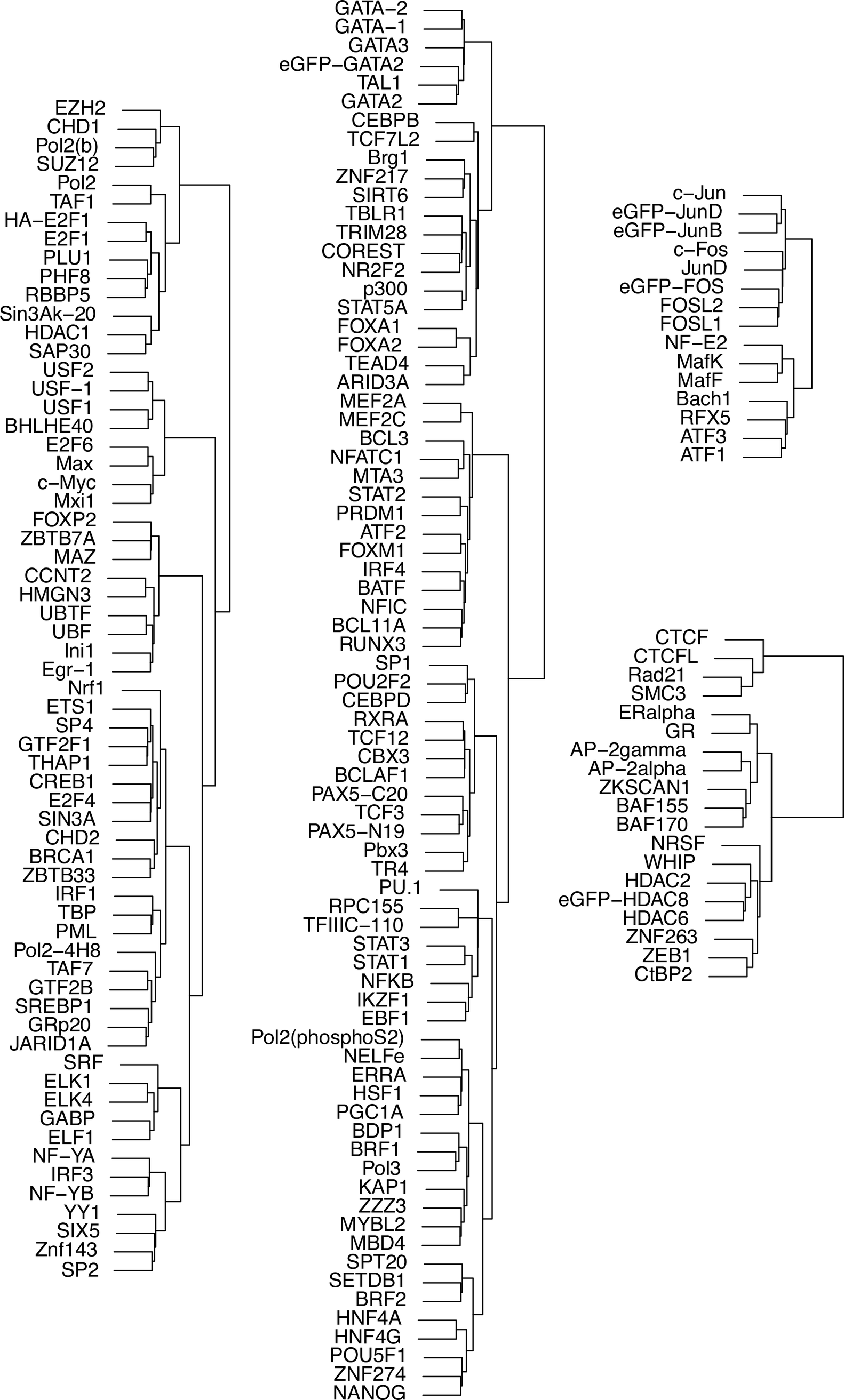

Supplement: S3 Fig — (TIF) [file pcbi.1005403.s008.tif]

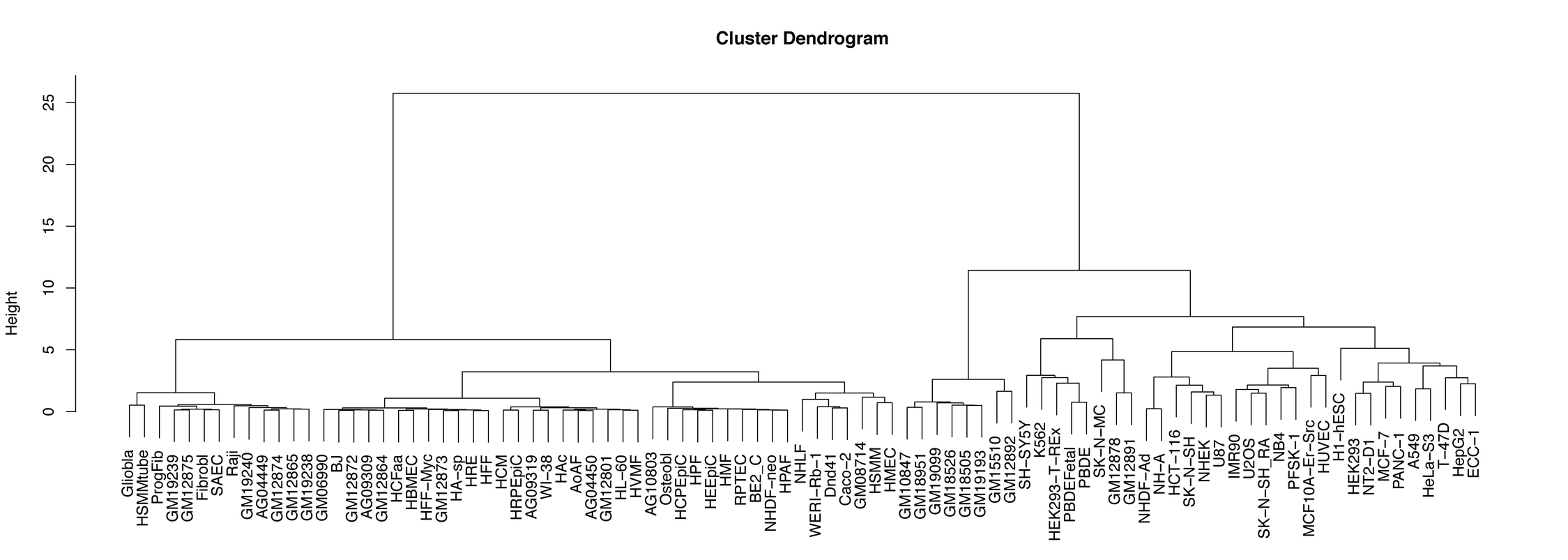

Supplement: S4 Fig — (TIFF) [file pcbi.1005403.s009.tiff]

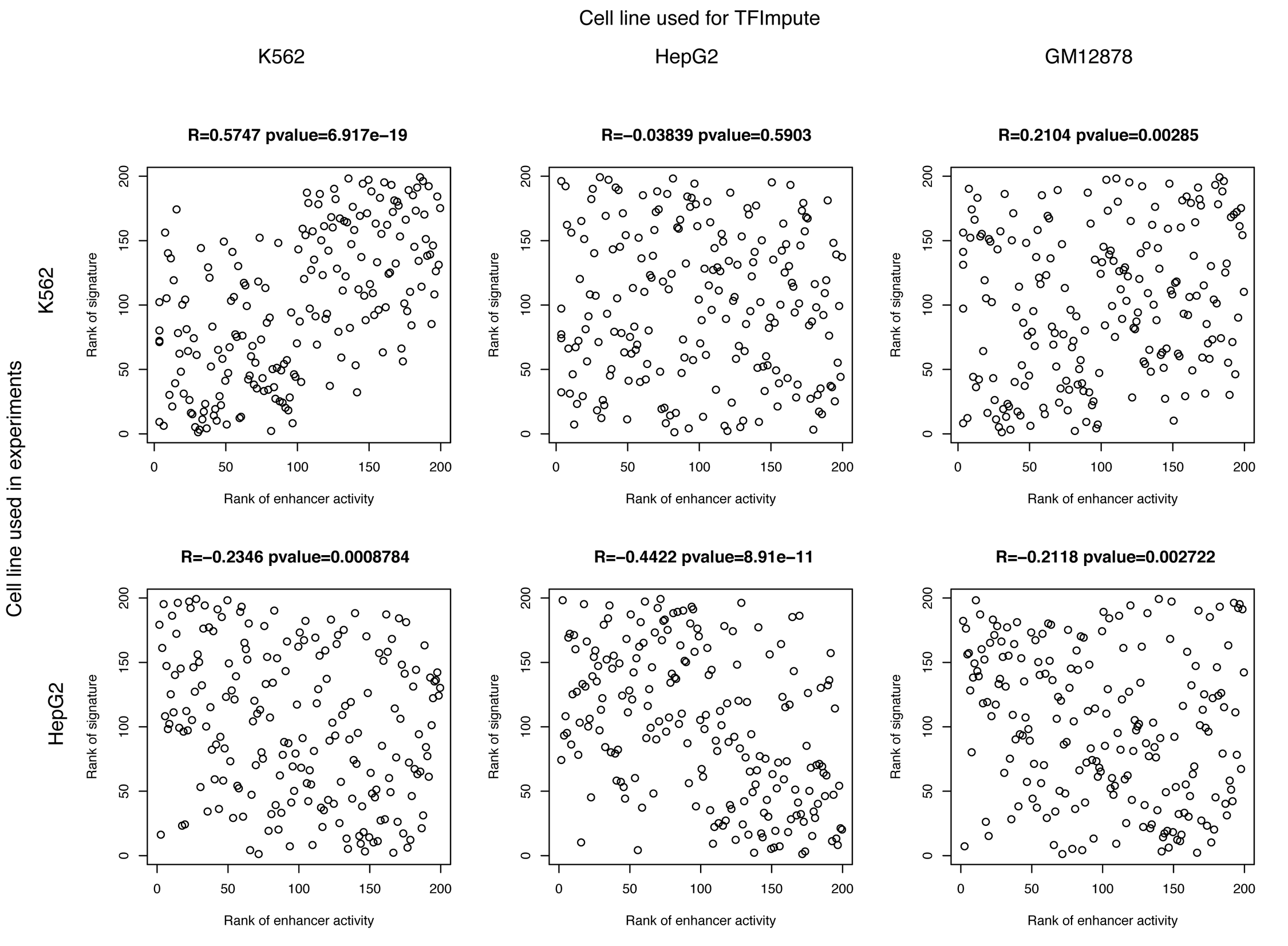

Supplement: S5 Fig — (TIFF) [file pcbi.1005403.s010.tiff]

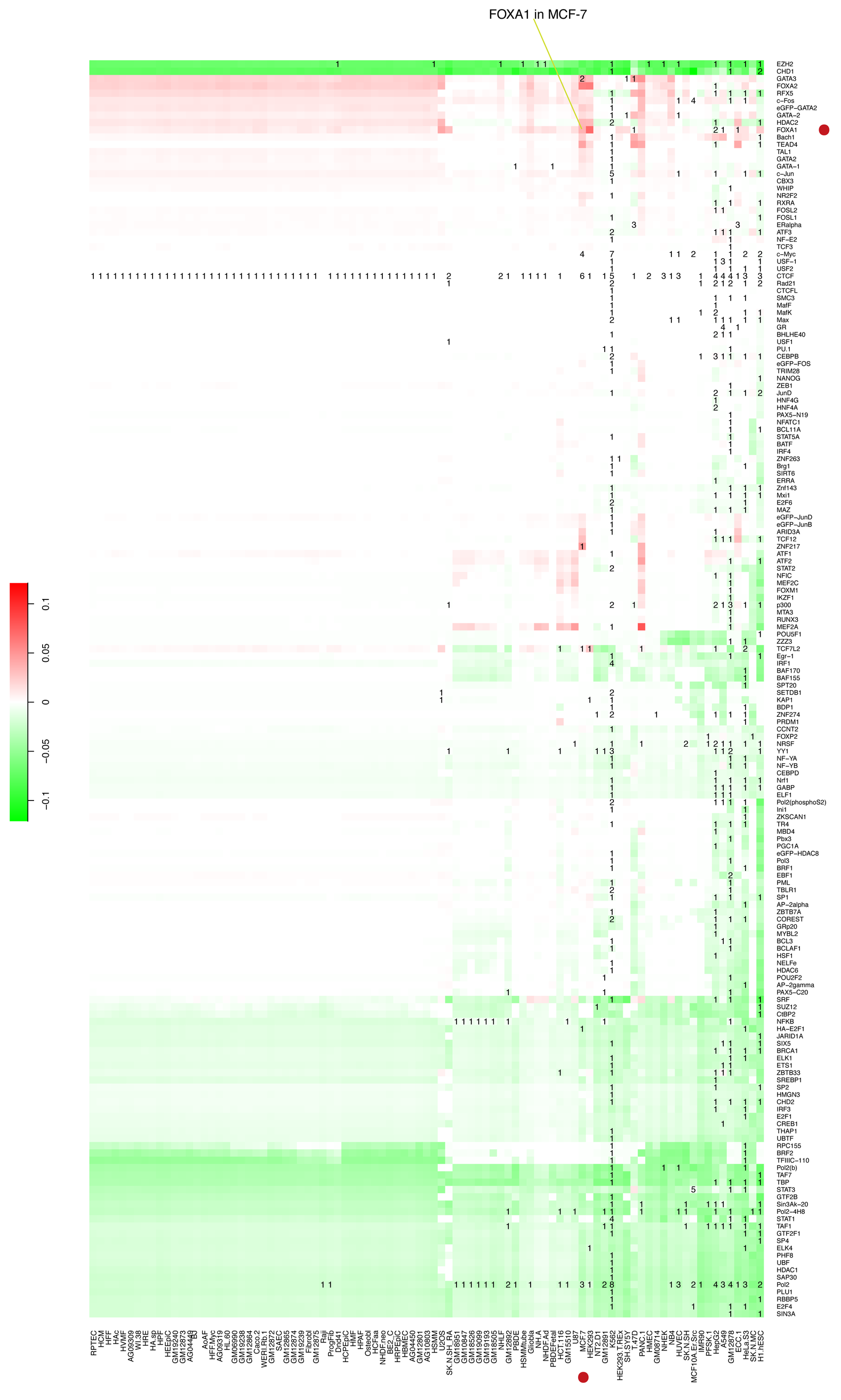

Supplement: S6 Fig — The color in each cell represents the predicted binding affinity of allele T minus that of allele C for the corresponding TF and cell line. (TIFF) [file pcbi.1005403.s011.tiff]

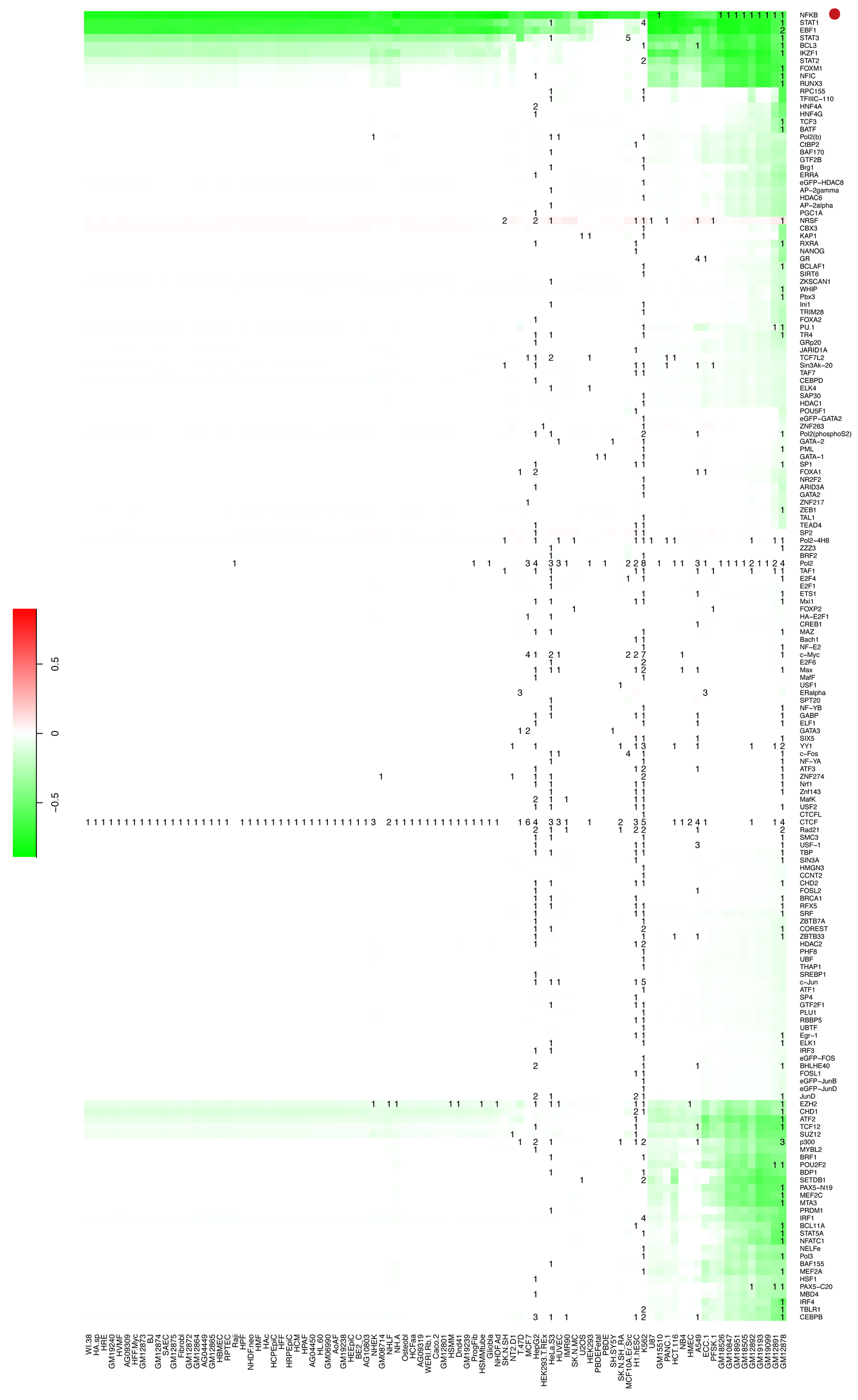

Supplement: S7 Fig — The color in each cell represents the predicted binding affinity of allele T minus that of allele C for the corresponding TF and cell line. (TIFF) [file pcbi.1005403.s012.tiff]
